# Sequence‐Defined Peptoids with —OH and —COOH Groups As Binders to Reduce Cracks of Si Nanoparticles of Lithium‐Ion Batteries

**DOI:** 10.1002/advs.202000749

**Published:** 2020-08-05

**Authors:** Qianyu Zhang, Chaofeng Zhang, Wenwei Luo, Lifeng Cui, Yan‐Jie Wang, Tengyue Jian, Xiaolin Li, Qizhang Yan, Haodong Liu, Chuying Ouyang, Yulin Chen, Chun‐Long Chen, Jiujun Zhang

**Affiliations:** ^1^ School of Materials Science and Engineering Dongguan University of Technology Dongguan Guangdong 523808 China; ^2^ Physical Sciences Division Pacific Northwest National Laboratory Richland WA 99352 USA; ^3^ Institutes of Physical Science and Information Technology Anhui University JiuLong Rd Hefei Anhui 230601 China; ^4^ Key Laboratory of Structure and Functional Regulation of Hybrid Material (Ministry of Education) Anhui University Hefei Anhui 230601 P. R. China; ^5^ Department of Physics Jiangxi Normal University Nanchang Jiangxi 330022 China; ^6^ Energy and Environmental Directorate Pacific Northwest National Laboratory Richland WA 99352 USA; ^7^ Department of NanoEngineering University of California San Diego La Jolla CA 92093 USA; ^8^ Department of Chemical Engineering University of Washington Seattle WA 98195 USA; ^9^ Institute for Sustainable Energy/College of Sciences Shanghai University Shanghai 200444 China

**Keywords:** capacity, lithium‐ion batteries, peptoids, polymeric binders, Si anodes

## Abstract

Silicone (Si) is one type of anode materials with intriguingly high theoretical capacity. However, the severe volume change associated with the repeated lithiation and delithiation processes hampers the mechanical/electrical integrity of Si anodes and hence reduces the battery's cycle‐life. To address this issue, sequence‐defined peptoids are designed and fabricated with two tailored functional groups, “—OH” and “—COOH”, as cross‐linkable polymeric binders for Si anodes of LIBs. Experimental results show that both the capacity and stability of such peptoids‐bound Si anodes can be significantly improved due to the decreased cracks of Si nanoparticles. Particularly, the 15‐mer peptoid binder in Si anode can result in a much higher reversible capacity (ca. 3110 mAh g^−1^) after 500 cycles at 1.0 A g^−1^ compared to other reported binders in literature. According to the density functional theory (DFT) calculations, it is the functional groups presented on the side chains of peptoids that facilitate the formation of Si−O binding efficiency and robustness, and then maintain the integrity of the Si anode. The sequence‐designed polymers can act as a new platform for understanding the interactions between binders and Si anode materials, and promote the realization of high‐performance batteries.

## Graphical abstract

The peptoids are programmed with the —COOH and —OH groups in precisely controlled numbers and distances acting as binders for Si anodes. These peptoids can not only facilitate the formation of Si—O binding efficiency and robustness, but also maintain the integrity of the Si anode, resulting in the increased active interaction between polymers and Si.

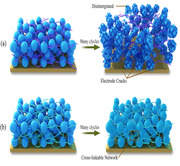

## 1 Introduction

As one of the most important materials for energy storage and conversion applications, Si with a high theoretical capacity of 4200 mAh g^−1^ has been extensively explored in the development of high energy/power density and long cycle‐life lithium‐ion batteries (LIBs).^[^ ^1a–f^ ^]^ Unfortunately, as an LIB anode materials, Si often suffers from a massive volume change (300%) during the repeated lithium insertion/extraction in battery charge/discharge processes, leading to severe particle pulverization, electrical contact loss, uncontrollable growth of solid electrolyte interphase (SEI), and subsequent degradation of the battery performance.^[^ ^2a,b^ ^]^ To address these issues, various natural and synthetic polymers have been explored and developed as binders to extend the cycle‐life of Si anodes.^[^ ^3a–f^ ^]^ Compared to the natural polymer binders, the synthetic polymer binders have some advantages such as the structure‐optimized design and the functionality‐tuned ability for achieving high‐performance Si electrodes. The obtained conductive and self‐healing polymer binders can enhance both the mechanical strength and electrical integrity of Si electrodes.^[^ ^4a–d^ ^]^ For example, using 2% functional conductive polymeric binder in micron‐size SiO particles‐based LIB anode, Zhao et al.^[^ ^5^ ^]^ obtained a capacity as high as 1000 mAh g^−1^ with ≈500 cycles. It seemed that the mechanical strength of such an anode material needed to be further improved. Wang et al.^[^ ^6^ ^]^ synthesized a self‐healing polymer binder for modifying Si microparticle (SiMP) anodes and achieved an excellent cycle life with a high capacity of ≈3000 mAh g^−1^. It seemed that more attention might be paid to the improvement of physicochemical properties of such an anode for improving initial columbic efficiency and irreversible capacity. Generally speaking, it is still a significant challenge to develop effective and multifunctional binders that can fully eliminate the severe volume change of anode materials during LIB charge/discharge processes.

In the effort to reduce the volume change of LIB Si‐based anodes, tailoring the structure of binders in the molecular level has become one of the efficient ways to alter their chemical and/or physical properties and enrich their functionalities. Particularly, an ideal binder for Si‐based anode should not only have strong adhesion but also elastomeric property that enables the Si to maintain the integrity during severe volume changes and relax back to their original state during lithiation/delithiation processes.^[^ ^7a–c^ ^]^ According to the reported literature, the binder containing carboxyl (—COOH) and hydroxyl (—OH) could form strong hydrogen bonding and ion‐dipole interaction with silanol groups of the native oxide layer formed on the Si surface, offering both the designed adhesion and elastic properties to the surface of Si anode.^[^ ^8a–d^ ^]^ Typically, carboxymethyl cellulose (CMC), poly[acrylic acid] (PAA), and poly[vinyl alcohol] (PVA), which contain both —COOH and —OH groups, have shown better efficiencies in constructing the durable interactions between binder and Si anode when compared to those non‐functional polymers such as PVDF which does not contain —COOH and —OH groups.^[^ ^9a–g^ ^]^ But, if the concentration of —OH group exceeds a certain value, the mechanical strength will not further increase because the adhesion is increased linearly with the number of —OH group to a maximum at about 27 OH per 100 carbon atoms.^[^ ^10^ ^]^ On the other hand, the adhesion and tensile strength of the polymers is decreased gradually with increasing ratio of —COOH/—NH_2_ in the binder molecule.^[^ ^11a,b^ ^]^ For precisely tuning the distribution and numbers of functional —COOH and —OH groups, it is still needed to give more effort to reveal the underlying interaction mechanism between the binding polymer and the Si particles, which can provide an effective and facile guide principles for the synthesis of more effective binders. In comparison with other types of polymer, the bio‐derived polymers, such as alginate, chitosan, gums, and carrageenan, have also been identified as the more effective binders for Si‐based anodes due to their richer —COOH and —OH groups.^[^ ^12a–e^ ^]^

In the exploration of effective polymer binders, we have found that sequence‐defined peptoids can work as the effective binders for LIB Si anodes although there is no related literature report about its usage as a binder. As one type of the sequence‐defined synthetic polymers, peptoids are highly stable with a narrow polydispersity index of molecular weight, whose side‐chains can be programmed with the controlled density, distance and arrangement of both —COOH and —OH groups.^[^ ^13a–c^ ^]^ In this work, we strategically have fabricated the sequence‐defined peptoids through a facile sub‐monomer synthetic method.^[^ ^14a–c^ ^]^ When peptoids are programmed with —COOH and —OH groups with precisely controlled numbers and distances, and then acted as binders for Si anodes, the electrochemical stability of the corresponding LIBs are effectively improved, not only because of the increased active interaction between polymers and Si but also the maintained integrity of Si electrode. The effects of the number and distance of —COOH and —OH functional groups on performance of Si anode have also systematically investigated and revealed the interaction mechanism between polymer binders and Si material. The fundamental understanding in this work can provide a new insight into the design of polymeric binders for enhancing electrochemical performance of LIB Si anodes.

## 2 Results and Discussion

In general, polymers as binders with highly polar groups, like —COOH and —OH, can form strong hydrogen bonds with Si, maintain electronic connection between Si and the binder, and improve the performance of Si electrodes.^[^ ^15a–c^ ^]^ The choice of peptoids as binders rely on the fact that it has the same functional groups that are present in bio‐derived polymers, that is, —COOH, —OH, —NH_2_, and —CH_3_. Long‐sequence‐defined peptoid can result in self‐assembly, which offers easy tunability, and the adjacent segments of peptoid can slide with respect to one another to offer externally‐triggered structural dynamics. However, in contrast, short‐sequence‐defined peptoid can avoid self‐assembly because it has fewer intermolecular hydrogen bonds than the longer one.^[^ ^15b^ ^]^ Moreover, this short‐sequence peptoid can be produced with high yield, which is low‐cost and chemically stable compared with the long‐sequence one.^[^ ^16a,b^ ^]^ Therefore, we have chosen a short‐sequence‐defined peptoid as binder for nano‐Si electrode. In this work, two 15‐mer peptoids (Figure S1, Supporting Information) containing —COOH and —OH/—OCH_3_ are used in Si‐binder system to examine their influence on adhesion. As shown in Figure S1a, Supporting Information, the peptoid‐1 (P1) contains rich functional groups of —COOH and —OH, which is expected to be favorable for cross‐linking and interaction with active materials. To elucidate the function of the —OH group, —OCH_3_ (Marked as —OMe in Figure S1b, Supporting Information) is introduced to substitute —OH groups, which is denoted as P2. To do so, two parameters, the cohesive energy density (CED) and solubility parameters (*δ*), are used here. The CED, widely used to estimate the mutual solubility, can be derived from the solubility parameters (*δ*_i_) using the following equation:

(1) $$\mathrm{CED}=\delta^{2}=\delta_{d}^{2}+\delta_{p}^{2}+\delta_{h}^{2}$$

where *δ_d_*, *δ_p_*, and *δ_h_* are the solubility parameters due to dispersion force, dipole force and hydrogen bonding, respectively.^[^ ^17a,b^ ^]^ As identified, the *δ_h_* had the highest positive impact and *δ_d_* had the lowest positive impact on CED.^[^ ^18^ ^]^ Therefore, it is imperative to put much more emphasis on Parameter *δ_h_* to predict the adhesive property of the peptoid type.

For a deeper understanding of the interaction mechanism and sorting out the key functional group that dominates the adsorption with the Si material, density functional theory (DFT) calculations are carried out to quantify the adsorption strength of the functional groups on the peptoids. In our simplified model, three molecules containing typical functional groups presented on the peptoids, —OCNCCO—, —COOH, and —OH, are selected for study and denoted as Molecule‐1, molecule‐2, and Molecule‐3, respectively. The optimized structures of the molecules at vacuum state are presented in Figure S1, Supporting Information.

The bonding pathways of the three molecules with Si anode surface were modeled with an eight‐layer slab model of the Si‐(111) surface (the computational details are shown in Supporting Information for more details). Different adsorption sites and configurations were investigated by changing the positions and orientations of the three molecules at the Si‐(111) surface, and we found that O atoms in both “—COOH” and “—OCNCCO—” functional groups could chemically bind with the Si‐(111) surface, while no chemical bonding of O atoms in “—OH” was observed. In order to quantitatively decide the bonding strength, the adsorption energy (*E_ad_*)can be defined by Equation 2:

(2) $$E_{ad}=E_{\mathrm{slab}}+E_{\mathrm{molecule}}-E_{\mathrm{slab}+\mathrm{molecule}}$$

where *E*_slab_ *, E*_molecule_, and *E*_slab+molecule_ are the calculated ground state energies of the clean Si‐(111) slab, the gas phase molecule, and the Si‐(111) slab adsorbed with the molecules, respectively. Table S1, Supporting Information presents the calculated adsorption energies of the three molecules on Si‐(111) with different adsorption configurations.

In our work, we have demonstrated the chemical bonds between nano‐silicon and sequence‐defined peptoid as binders for the first time, which can buffer the volume changes in the silicon electrode. Unlike those reported binder systems for the silicon anodes with interactions such as hydrogen bonding interactions (SiOH‐HC) or covalent bonding between the Si and the binder (SiO‐OC), our condensation bonding between the silicon and binder has the highest binding energy for facilitating the stability of the electrode.

**Figure 1:**
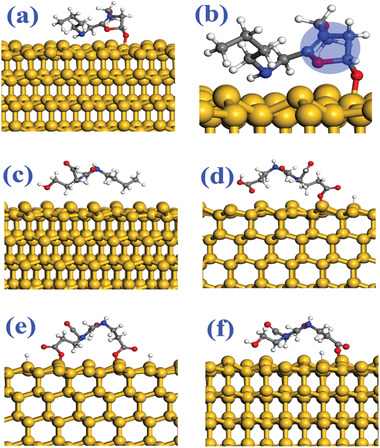
Adsorption configurations of the three molecules on Si‐(111) surface. a,b) Molecule‐1 with one O atom from the —OCNCCO— group bonded to Si atom, c) physical adsorption of Molecule‐1, d,e) Molecule‐2 with one and two O atoms from —COOH group bonded with Si atom, and f) Molecule‐3 with one O atom from —COOH group bonded with Si atom. The yellow, red, gray, blue, and white spheres are Si, O, C, N, and H atoms, respectively. The C—N—C—C—O penta‐ring is highlighted with a transparent blue cycle.

In order to better understand the interactions between silicon and binders, three molecules are further constructed to simulate the condensation bonding between silicon and the binder. Their optimized structures are shown in **Figure** **1**. The values oft he binding energy, *E*_b_, of the three molecules, which correspond to the strength of bond breaking, are calculated using the following Equation 3:^[^ ^2b^ ^]^

(3) $$\begin{array}{ccc} E_{b}\left(M_{x}-\mathrm{Si}\right) & = & \left[E_{\mathrm{tot}}\left(M_{x}\right)+E_{\mathrm{tot}}\left(\mathrm{Si}\right)-E_{\mathrm{tot}}\left(M_{x}-\mathrm{Si}\right)\right]\\ & ÷ & A\left(M_{x}-\mathrm{Si}\right) \end{array}$$

Where *E*_tot_(M_*x*_), *E*_tot_(Si), and *E*_tot_(M_*x*_ − Si) are the total energies of the relaxed M*_x_*, Si and M*_x_*‐Si models, respectively. Herein, *x* = 1, 2, and 3 representing M1, M2, and M3 molecule models, respectively. *A*(M_*x*_ − Si) is the surface area of the relaxed M_*x*_ − Si model. The binding energies *E*_b_ of M1, M2, and M3 molecule models are calculated to be 2.428, 1.175, and 0.349 eV nm^−2^, respectively. The high binding energy in our work can explain the interaction between Si and the P*_x_* (*x* = 1, 2, 3) binder to some extent. Furthermore, the adsorption energy of the molecules on Si‐(111) was strongly dependent on the adsorption configuration, as shown in Table S1, Supporting Information, particularly upon the formation of Si—O chemical bonding. Without formation of such a chemical bonding, the adsorption energy is close to 1 eV. Once Si—O bond is formed between the molecule and the Si‐(111) surface, the adsorption energy is increased to ≈3.5 eV. Furthermore, as Molecule‐2 contains two “—COOH” groups, two Si—O bonds can be formed and the adsorption energy reaches to 5.54 eV. These results further verify that the peptoids enriched with —COOH groups have very strong binding interactions with Si anode surface.

Since the strong adhesion of the peptoids to the conductive carbon is also very critical, the adsorption energy of the three molecules to the graphene layer is about 0.8 eV for each molecule, as presented in Table S1, Supporting Information. As the conductive carbon does not show drastic volume change during the charge/discharge process, we assume that the above adsorption energy should be sufficient for the steady bonding of the peptoid binder with the conductive carbon. According to aforementioned theoretical calculations, the stabilization mechanism of nano‐Si electrodes based Peptoid 1 (P1) binder can be demonstrated in Scheme S1, Supporting Information.

**Figure 2:**
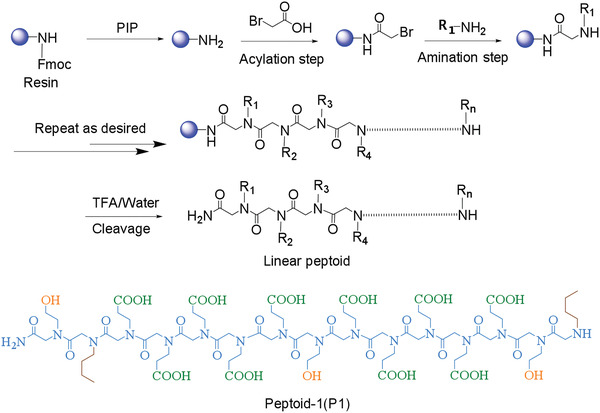
Schematic of the peptoid solid‐phase submonomer synthesis and the structure of P1.

Solid‐phase submonomer synthesis (SPSS) was also used to directly synthesize sequence‐specific biopolymers on inert solid‐support such as a polymeric resin bead.^[^ ^19a,b^ ^]^ This method can give high coupling yields and ease removal of excessive reactants. The typical SPSS process and the molecular structure of Peptoid‐1 (P1) are illustrated in **Figure** **2**. The excessive reagents can be simply drained and the beads can be washed for the next reaction step after a coupling reaction with the resin. Then, the full‐length oligomers are cleaved from the resin, and the solution‐phase material can be further investigated.

Ultra‐Performance Liquid Chromatography (UPLC) spectra for purified P1 and P2 are shown in Figures S3 and S4, Supporting Information. Their molecular weights are 1839.14 and 1880.02 (Found: [M/2+H] ^+^), respectively. For P1 binder, the well‐designed peptoid sequences with —COOH and —OH functional groups are compatible with Si materials and additives. Besides, —(CH_2_)_3_CH_3_ group can be incorporated into the sequence to modulate peptoid hydrophobicity and facilitate the purification. The dual functional groups are expected to show better affinity towards the polar hydroxyl groups on the Si surface. More importantly, they can enable thermal crosslinking through etherification reaction. By carefully programming the —COOH and —OH groups in terms of their number and position in the sequences, the peptoids can be crosslinked into a cage‐like network, confining the Si particles inside. Given the robust and flexible polymeric networks, such a configuration with Si anode should be very promising to accommodate the drastic volume expansion and contraction during charge/discharge processes, leading to high capacity efficiency and retention.

FT‐IR is a powerful tool for revealing the chemical bonds. Successful synthesis of P1 and P2 binders was further confirmed by comparing their FT‐IR spectra with pure CMC, as shown in Figure S5, Supporting Information. In the spectra of P1 and P2, the peak at 1715 cm^−1^ can be attributed to C=O in carboxylic acid.^[^ ^20^ ^]^ A sharp peak at 1177 cm^−1^ is ascribed to a C—OH stretching mode.^[^ ^21^ ^]^ These two peaks verify the existence of the carboxyl groups in P1 and P2. The absorption peaks at 1638 and 1404 cm^−1^ belong to the —CO—N— groups,^[^ ^22^ ^]^ which do exist in the modules of these two peptoids. A broad absorption band at 2922 cm^−1^ is ascribed to the stretching of C—H.^[^ ^23^ ^]^ Additionally, the peak at 1134 cm^−1^ corresponds to the functional group of —C‐OCH_3_ in P2, and the broad peak observed at 3418 cm^−1^ is assigned to the O—H stretching in P1. As for the pure CMC, the observed two peaks at 1413 and 1588 cm^−1^ correspond to the antisymmetric stretching and symmetric stretching of COO^−^, respectively, which are consistent with the previous studies.^[^ ^20^, ^21^, ^22^, ^23^ ^]^

**Figure 3:**
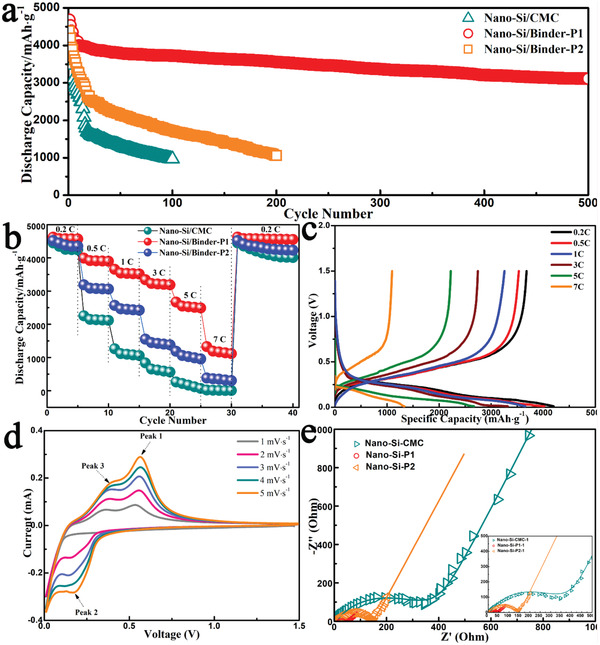
Electrochemical performance of nano‐Si anodes with CMC, P1, and P2 binders. a) Cycling Performance of nano‐Si based electrodes containing CMC, P1, and P2 binders at a current density of 1 A g^−1^. b) Rate capability of nano‐Si electrodes at different current densities between 0.2 and 7 C rates. c) Charge‐discharge curves of P1‐based nano‐Si electrodes at various current rates. d) Cyclic voltammetric curves of P1‐based nano‐Si anodes at different scan rates from 1 to 5 mV s^−1^. e) EIS spectra of nano‐Si anodes with CMC, P1, and P2 as binders.

The effectiveness of the binders was also examined by electrochemical tests. **Figure** **3** shows the electrochemical performances of the Si anodes prepared using CMC, P1, and P2 as binders. As shown in Figure 3a, Si anodes with P1 binder exhibit a significantly improved cycling performance when compared with CMC binder. In particular, the Si anode with P1 binder can deliver the highest capacity among all three samples, that is, a high capacity of 3111.0 mAh g^−1^ can be retained after 500 cycles under a current density of 1 A g^−1^. And the anode also shows a more superior cycling performance when compared to previously reported Si anodes (as listed in Table S2, Supporting Information). The capacity decay in the first few cycles can be ascribed to the increased electrolyte penetration/electrode activation during battery operation.^[^ ^24^ ^]^ In the first few cycles, the binder provides suitable binding interactions with both the active material and the conductive additive to form a stable thin SEI film on the silicon surface, which leads to a capacity loss during the initial cycles. Furthermore, the typical initial charge/discharge profiles of these binders‐based anodes at a current densities of 1 and 15 A g^−1^ between the potential ranges of 0.01–1.5 V are shown in Figures S6A and S6B, Supporting Information, respectively. The first discharge capacities of CMC, P1, and P2 based nano‐Si anodes are 4314, 4689, 4540mAh g^−1^ at 1 A g^−1^, corresponding to the Columbic efficiencies of 68.4%, 75.3%, and 72.8%, respectively. Although the Columbic efficiency of the electrode with P1 binder is only 75.3% for the first cycle, it is increased rapidly to 95.6% at the second cycle and then remained steady above 99% after 100 cycles. The possible reason for this phenomenon is that the P1 crosslinked binder can provide suitable binding interactions with both the active material and the conductive additive, leading to the formation of a stable secondary structure. It is worth to noted that when P2 is used as the binder, the Si anode shows a poorer cycling performance, indicating the presence of —OH functional groups is more favorable for the bonding between binder and Si.

The above comprehensive results prove that P1 binder outperforms CMC. CMC has a very similar structure to P1, however, it can only interact with electrode through weak Van de Waals forces due to the difficulty in precisely tuning over the functional groups, and eventually fails to suppress the drastic volume change and maintain integrity of the electrodes.^[^ ^9a–c^ ^]^ Unlike CMC, the peptoids have the programmed functional groups with precisely controlled composition and number. They contain rich functional groups, such as —COOH, —OH, and —(CH_2_)_3_CH_3_, which are also favorable in crosslinking and interaction with active materials. When implemented as binders for Si anodes, the presence of functional groups on the peptoids can increase adsorption between polymers and Si. This leads to the improved electrode integrity and cycling performance.

Figure 3b shows the rate capability of nano‐Si anodes with those three binders. The CMC based nano‐Si anode delivers the worst rate performance. The discharge capacities are 1261.0, 837.5, 266.2, and 21.5 mAh g^−1^ at the current rates of 1, 3, 5, and 7 C, respectively. The nano‐Si anodes with peptoids as binder shows a better rate capability than that of CMC. In particular, the P1 based nano‐Si anode exhibits the best rate capability. The discharge capacity can remain at 1328.2 mAh g^−1^ even at a high rate of 7 C. Upon returning back to 0.2 C, the capacity can be almost recovered to its initial value. Such a remarkable rate capability of nano‐Si anode with P1 binder can be attributed to the effective cross‐linking created by —COOH and —OH functional groups, which enables the effective inosculation of Si nanoparticles, super P conductive materials and current collector as a whole. The galvanostatic charge/discharge curves can reveal the electrochemical behavior of the P1‐based nano‐Si anode. It can be seen from Figure 3c that the nano‐Si anode with P1 binder exhibits a low polarization even at 5 C, which well agrees with the aforementioned best rate performance. To further investigate the effect of —COOH and —OH functional groups on the reaction kinetics of nano‐Si anodes, typical cyclic voltammetric (CV) curves of the anodes with CMC, P1, and P2 binders at a scan rate of 0.2 mV s^−1^ were recorded and the results are shown in Figure S6C, Supporting Information. It can be found that the nano‐Si anode with P1 binder has the smallest potential difference and the largest specific current response and, thereby, the highest rate capability. Figure 3d shows the CV curves of P1‐based nano‐Si anodes at different scan rates. One broad Li‐insertion peak appears at 0.21 V and two distinct Li‐extraction peaks are located at 0.37 and 0.58 V, which are in agreement with previously reported results.^[^ ^25^, ^26^ ^]^ The peak position is nearly unchanged as the scan rate raising from 1 to 5 mV s^−1^, indicating a good stability of P1‐based nano‐Si anodes.

In order to understand the superior cycling performance of P1‐based nano‐Si anodes, the measurements using electrochemical impedance spectroscopy (EIS) of the anodes with CMC, P1, and P2 binders, respectively, were conducted after 100th cycles at a current density of 1 A g^−1^. As shown in Figure 3e, each curve is composed of a depressed semicircle in the high‐frequency region and a straight line in the low‐frequency region. The intermediate‐frequency semicircle represents the charge‐transfer resistance (*R*_ct_) at the electrode/electrolyte interface, while the low‐frequency tail is associated with Li^+^ ion diffusion in Si nanoparticles. The impedance spectra were fitted as inset in Figure 3e. It can be seen that the nano‐Si anode with P1 binder has the smallest *R_ct_*, indicating that the Si nanoparticles can be strongly crosslinked as a mechanical integrity by —COOH and —OH functional groups in P1 binder, and thus minimize the contact impedance during the cycling.

The Li^+^ diffusion coefficient can be further calculated using the following equations:^[^ ^27a–c^ ^]^

(4) $$D_{Li^{+}}=\frac{1}{2}{\left(\frac{RT}{n^{2}F^{2}AC_{\mathrm{Li}}\sigma }\right)}^{2}$$

(5) $$Z_{\mathrm{re}}=R_{\mathrm{ct}}+R_{s}+\sigma \omega^{-0.5}$$

where *R* is the gas constant, *T* is the absolute temperature, n is the number of electrons transferred in the half‐reaction for the redox couple, *F* is the Faraday constant, *A* is the geometrical electrode area, *C*_Li_ is the concentration of lithium ion in the solid, and *σ* is the Warburg factor. The slope of the *Z*_re_‐*ω* ^−0.5^ line in Figure S6D, Supporting Information shows that the nano‐Si anode using P1 binder has a much higher Li^+^ ion diffusion coefficient after cycling compared to the anodes with CMC and P2 as the binders. These results indicate that P1 binder with strong adhesion to Si nanoparticles could maintain a stable integrity, and thus improve the Li^+^ ion transport and the overall charge transfer resistance of nano‐Si anodes.

To verify our aforementioned results, we also carried out electrochemical tests on 39‐mer sequence‐defined peptoid (denoted as P3). Its chemical structure and UPLC‐MS data are shown in Figure S7, Supporting Information. Figure S8, Supporting Information compares the electrochemical performance of Si anodes prepared using CMC and P3 as the binders. Figure S8a, Supporting Information shows the first Li charge/discharge profiles of the Si anodes. The electrode using binder P3 exhibits an initial capacity of ≈650 mAh g^−1^, which is higher than that of the sample using CMC binder. More importantly, as shown in Figure S8b, Supporting Information, the anode using P3 binder demonstrates a capacity of ≈3000 mAh g^−1^ after 150 cycles at 3 C rate, which is much higher than ≈800mAh g^−1^ obtained in the Si anode using CMC as the binder. Figure S8c, Supporting Information shows the discharge capacity of the anodes at different discharge rates. The anode using P3 as the binder exhibits a much better high rate capability than that using CMC binder. For example, at 3C rate, P3‐based anode can retain a capacity of ≈2000 mAh g^−1^. Figure S8d, Supporting Information compares the capacities of anodes using CMC and P3 as the binders. The high rate capability of Si anode using P3 binder is consistent with the anode impedance spectra shown in Figure S9a, Supporting Information, which can compare with the impedance spectra of the two anodes after three formation‐cycles. The anode using P3 binder exhibits an impedance of only about 25 Ohm as compared to ≈400 Ohm for the anode using CMC as the binder. Figure S9b, Supporting Information shows the CVs of those two anodes. The sample with P3 exhibits both higher reduction and oxidation peaks as compared with those using CMC binder. These results further indicate that P3 can enhance both the electronic (Figure S9b, Supporting Information) and ionic (Figure S9a, Supporting Information) conductivities of the Si anode. However, the results are not as good as aforementioned anode with P2 as the binder. This may be attribute to the self‐assembly when the sequence becomes longer, which could affect the crosslinking in the peptoid.

**Figure 4:**
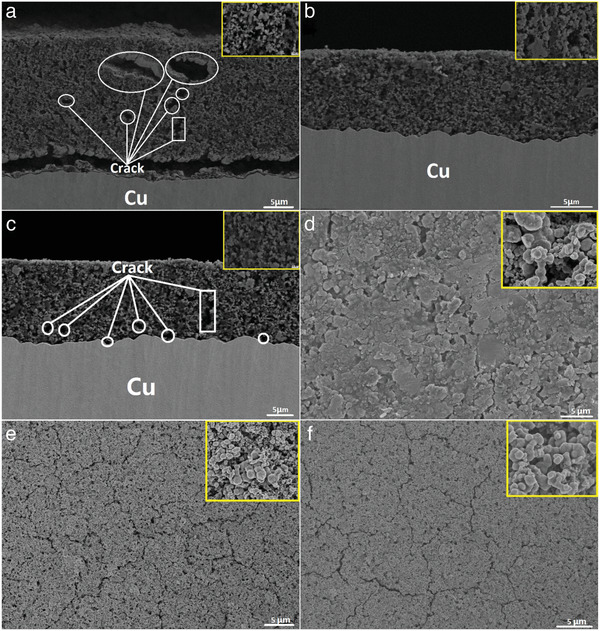
Cross‐sectional SEM images of nano‐Si anodes with A) CMC, B) P1, and C) P2 as binders before cycling, separately. The inset in (A), (B), and (C) are magnified images. (D), (E), and (F) are the top‐viewed SEM images of A) CMC, B) P1, and C) P2 bound nano‐Si anodes before cycling, respectively. The inset in (D), (E), and (F) are their partly magnified images.

Ion milling technology was also used to investigate the morphological and structural changes during cycling of the anodes. The mass loadings of A) CMC, B) P1, and C) P2 based Nano‐Si electrodes are ≈1.5 mg cm^−2^. We also measured the thicknesses of the electrode layers (**Figure** **4**), showing that A is ≈20 micron, B is ≈12 micron, and C is ≈15 micron, respectively. At pristine state, the cross section of the P1‐based electrode is much thinner than that of CMC (Figure 4).

Figure 4A–C are the cross‐sectional SEM images of nano‐Si anodes with CMC, P1, and P2 as binders before cycling. It can be clearly seen from Figure 4B that no obvious cracks can be found for the nano‐Si anode with P1 binder, whereas the ones with CMC and P2 as the binders show obvious microcracks seen from Figure 4A,C. As shown in Figure 4A–C, many cracks and/or large pores appear in the electrode of nano‐Si with CMC as a binder, while only very few cracks and/or pores can be found in the nano‐Si with P1 binder. The main reason is that the interactions between binder CMC and Si or Cu are weaker than those between P1 and Si or Cu during the heating process. The water in the slurry containing Si, CMC, P1, and carbon will evaporate away during heating process, which will make the electrode of Si‐CMC thicker than the electrode of Si‐P1 due to the weaker interaction in CMC—Si—Cu. On the other hand, the insufficient cohesive force to active materials and Cu foil suggests that the Nano‐Si electrode with CMC binder has a poor mechanical capacity to tolerate the stress induced by heating in the oven. Therefore, there is more porosity in the electrode with CMC than that of P1 and P2. In additional, nano‐Si anode using P2 binder demonstrates smaller and less cracks than CMC binder, suggesting that —COOH functional groups are beneficial to enhancement of the adhesion. However, P2‐bound anode exhibits worse adhesion than P1, indicating that —OH plays a critical role in the cohesion of the electrode components and the adhesion to Cu current collector. The inserted images shown in Figure 4A–C also demonstrate the consistent results. The cohesion between Si nanoparticles for P1‐bound electrode exhibits an intimate adhesion, whereas CMC and P2 bound nano‐Si do not coalesce so well. The top‐viewed images further unveil their surface morphologies. As shown in Figure 4D–F, the P1 binder keeps the Si nanoparticles together without serious disintegration (Figure 4E). However, CMC‐based electrode shows some severe aggregated Si clusters (Figure 4D) and there are deep and long cracks on the surface of P2‐bound electrode (Figure 4F). Only the P1 binder can firmly anchor onto the nano‐Si surface via the —OH functional groups.

The P1‐bound electrode monitored by SEM (Figure S10B, Supporting Information) clearly shows that the morphology of nano‐Si anode is well preserved after cycling, whereas the ones with CMC and P2 as the binders show deep microcracks over 100 cycles (Figure S10A,C, Supporting Information), suggesting that nano‐Si anode with P1 as the binder has the highest mechanical strength to tolerate the stress induced by severe volume changes in Si nanoparticles during the charge–discharge cyclic processes. The enhanced adhesion between binder and Si nanoparticles can be attributed to the network structure formed by P1's adhesive groups, which are “—OH,” “—COOH,” and “—OCNCCO—” functional groups. Both the —COOH and —OH groups are expected to form strong supramolecular interactions with Si, which can dissipate the mechanical stress from the volume change of Si during the repeated charge and discharge cycles. According to SEM observation, the stabilization mechanism of nano‐Si anodes with P1 as the binder can be demonstrated in Scheme S2, Supporting Information.

**Figure 5:**
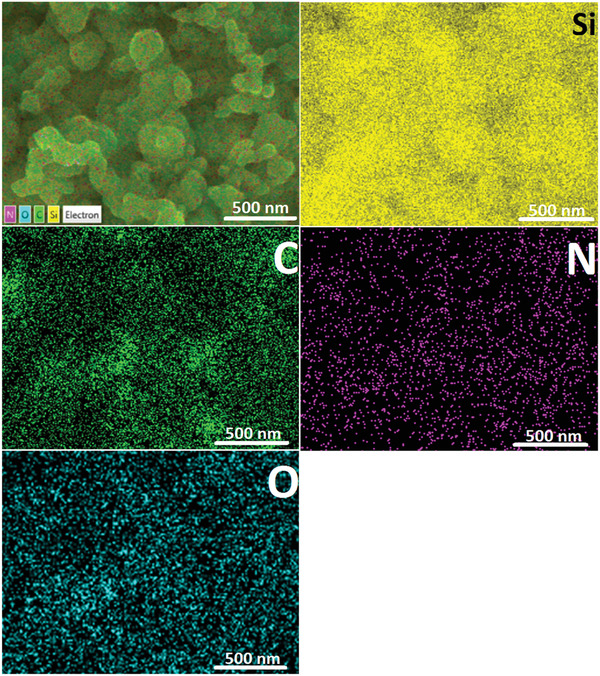
Elemental distribution information of the selected area on the P1‐bound nano‐Si anode after 100 cycles.

To investigate the compositional and structural features, elemental distribution scanning was conducted on the P1‐bound nano‐Si anode. It can be clearly seen from **Figure** **5** that the silicon, carbon, nitrogen, and oxygen elements can uniformly distribute in P1‐bound anode even after 100 cycles. More information of these elements can be obtained from the EDS spectrum in Figure S11, Supporting Information and elemental composition in Table S3, Supporting Information. X‐ray diffraction (XRD) was also used to characterize the bound Si anodes after 100 cycles. As shown in Figure S12, Supporting Information, the main diffraction peaks for CMC, P1, and P2 bound Si anodes at 2*θ* = 28.4°, 47.4°, and 56.0° can be indexed to be the reflections of the (111), (220), and (311) planes of Si crystallites (JCPDS No. 27–1402), respectively.^[^ ^26^, ^27^ ^]^ The coexistence of Cu element comes from the current collector used for XRD analysis. X‐ray photoelectron spectroscopy (XPS) analysis was carried out to further understand the effect of the binders on Si anode surface. As shown in Figure S13, Supporting Information, the C 1s spectra of CMC, P1, and P2 display peaks at 288.3, 286.7, and 284.4 eV, which correspond to the carbon in —COOR, C—O, and C—C bonds, respectively. The XPS data shows that there are no any obvious changes of the chemical structure after 100 cycles.

## 3 Conclusion

In this work, the sequence‐defined peptoids as cross‐linkable polymeric binders with two tailored and optimized functional groups, “—OH” and “—COOH”, are successfully designed and fabricated, and used as the binders for Si‐based anodes of lithium‐ion batteries (LIBs). SEM images prove that the presence of —OH and —COOH groups in such peptoids can effectively reduce the cracks of the Si nanoparticles during the charge/discharge processes of LIB. This is because such a peptoid (P1) can firmly anchor onto the nano‐Si surface via the optimized design of —OH and —COOH groups. XRD and XPS results demonstrate that compared to CMC and P2, the P1‐based Si anode has no obvious crack after long‐term cycles, suggesting that P1 can deliver better structural and chemical stability for Si anode. According to electrochemical measurements, P1‐bound Si anode can give the highest reversible capacity of 3110 mAh g^−1^ for 500 cycles at a current density of 1.0 A g^−1^, indicating that the usage of P1‐as the binder can induce the improved stability, maintain the integrity of Si nanoparticles, and enhance the electrochemical performance. For fundamental understanding, the density functional theory (DFT) calculations are carried out, and the results demonstrate that when the peptoids are programmed with precisely controlled numbers and distances of —COOH and —OH groups for binder of Si anodes, these functional groups presented on the side chains of peptoids can not only facilitate the formation of Si—O binding efficiency and robustness, but also maintain the integrity of the Si anode, resulting in the increased active interaction between polymers and Si and thereby the enhanced electrochemical performance. This work should provide an effective and facile guide principle to the development of more effective polymer binders for high‐capacity Si electrodes in LIBs.

## Conflict of Interest

The authors declare no conflict of interest.

## Supporting information

Supporting Information
