# Supplementary material for: Sequence‐Defined Peptoids with —OH and —COOH Groups As Binders to Reduce Cracks of Si Nanoparticles of Lithium‐Ion Batteries
Source: Adv Sci (Weinh). 2020 Aug 5;7(18):2000749. doi: 10.1002/advs.202000749 (PMC7509666; doi:10.1002/advs.202000749)
Supplement: Supplementary file 1 — Supporting Information [file ADVS-7-2000749-s001.pdf]

© 2020 Wiley-VCH GmbH

ADVANCED SCIENCE
Open Access

Supporting Information

for *Adv. Sci.*, DOI: 10.1002/advs.202000749

Sequence-Defined Peptoids with –OH and –COOH Groups As Binders to Reduce Cracks of Si Nanoparticles of Lithium-Ion Batteries

Qianyu Zhang, Chaofeng Zhang, Wenwei Luo, Lifeng Cui, Yan-Jie Wang, Tengyue Jian, Xiaolin Li, Qizhang Yan, Haodong Liu, Chuying Ouyang, Yulin Chen, Chun-Long Chen,* and Jiujun Zhang**

Sequence-defined Peptoids with -OH and -COOH Groups as Binders to Reduce Cracks of Si Nanoparticles of Lithium-Ion Batteries

*Qianyu Zhang, Chaofeng Zhang, Wenwei Luo, Lifeng Cui, Yan-Jie Wang**, Tengyue Jian, Xiaolin Li, Qizhang Yan, Haodong Liu, Chuying Ouyang, Chun-Long Chen**, *Jiujun Zhang**

Prof. Q. Y. Zhang, Prof. L. F. Cui, and Prof. Y. J. Wang
School of Materials Science and Engineering, Dongguan University of Technology, Dongguan 523808, China
E-mail: wyj@dgut.edu.cn (Prof. Y.-J. Wang);
Prof. Q. Y. Zhang, Dr. T. Y Jian and Prof. C.-L. Chen
Physical Sciences Division, Pacific Northwest National Laboratory, Richland, WA 99352, USA
E-mail: chunlong.chen@pnnl.gov (Prof. C.-L. Chen)
Prof. J. J. Zhang
Institute for Sustainable Energy/College of Sciences, Shanghai University, Shanghai 200444, China
E-mail: jiujun@shaw.ca (Prof. J. J. Zhang)
Prof. X. L. Li
Energy and Environmental Directorate, Pacific Northwest National Laboratory, Richland, WA 99352, USA
Dr. H. D. Liu and Q. Z. Yan
Department of NanoEngineering, University of California San Diego, La Jolla, CA 92093, USA
Prof. C. F. Zhang
Institutes of Physical Science and Information Technology, Anhui University, JiuLong Rd, Hefei 230601, China
Key Laboratory of Structure and Functional Regulation of Hybrid Material (Ministry of Education), Anhui University, Hefei, Anhui 230601, P. R. China
W. W. Luo and Prof. C. Y. Ouyang
Department of Physics, Jiangxi Normal University, Nanchang 330022, China
Prof. C.-L. Chen

Department of Chemical Engineering, University of Washington, Seattle, WA, 98195 USA

Chemical agents and materials

β-alanine tert-butyl ester hydrochloride purchased from Chem-Impex International, Inc. was deprotected by sodium hydroxide aqueous solution, extracted with CH_2Cl_2, then filtered and rotary evaporated for further reaction. *N,N'*-diisopropylcarbodiimide, bromoacetic acid and trifluoroactic acid (TFA) were purchased from Chem-Impex International, Inc. All other amine submonomers and reagents were purchased from commercial sources and used without further purification.

Carboxymethyl cellulose (CMC) binder and Si powder with an average particle size of ~ 50 nm were purchased from Alfa Aesar chemical Inc. Acetylene black was obtained from Guangzhou Lithium Force Energy Co. (China). Celgard 2400 was employed as a separator. The electrolyte of 1 M $LiPF_6$ in ethylene carbonate (EC, ≥ 99.9%)/diethylene carbonate (DEC, ≥ 99.9%)/dimethyl carbonate (DMC, ≥ 99.9%) (Volume rate: 1/1/1, water content ≤ 20ppm) was purchased from GuoTai-HuaRong New Chemical Materials Co. (China). All these materials were used as received.

Automated solid-phase synthesis of peptoids

Peptoid-1 (P1), Peptoid-2 (P2) and Peptoid-3 (P3) were synthesized on a commercial Aapptec Apex 396 robotic synthesizer using a modified solid-phase submonomer synthesis method as described previously.[1a, b] Rink amide resin (0.09 mmol) was used to generate C-terminal amide peptoids. In this method, the Fmoc group on the resin was deprotected by

adding 2 mL of 20 % (v/v) 4-Methylpiperidine/N, N-dimethylformamide (DMF), agitatied for 20 minutes, drained, and washed with DMF. All DMF washes consisted of the addition of 1.5 mL of DMF, followed by an agitation for 1.5 minutes (repeated five times). An acylation reaction was then performed on the amino resin by an addition of 1.6 mL of 0.6 M bromoacetic acid in DMF, and followed by 0.35 mL of 50 % (v/v) N, N-diisopropylcarbodiimide (DIC)/DMF. The mixture was agitated for 30 minutes at room temperature, drained, and washed with DMF for 5 times. Nucleophilic displacement of the bromide with various primary amines was carried out by a 1.6 mL addition of the primary amine monomer as a 0.6 M solution in N-methyl-2-pyrrolidone (NMP), and followed by an agitation for 60 minutes at room temperature. The monomer solution was drained from the resin, and the resin was washed with DMF for 5 times. The acylation and displacement steps were repeated until the peptoid with the desired length was synthesized. Desired peptoids were cleaved from resin by adding 95 % trifluoroacetic acid (TFA)/water, and then dissolving into water and acetonitrile (v/v = 1 : 1) for further HPLC purification.

Purification of Peptoid

Petpoid crudes were purified by a reverse-phase HPLC on a XBridgeTM Prep C18 OBDTM column (10 μm, 19 mm ×100 mm), using a narrow gradient of acetonitrile in H_2O with 0.1 % TFA over 15 minutes. Purified peptoids were analyzed using Waters ACQUITY reverse-phase UPLC (the corresponding gradient at 0.4 mL/min over 7 minutes at 40°C with a

ACQUITY®BEH C18, 1.7 μm, 2.1 mm × 50 mm column) that was connected with a Waters SQD2 mass spectrometry system. The final peptoid product was lyophilized from its solution in a mixture (v/v = 1:1) of water and acetonitrile. The lyophilized peptoid powder was then used for battery test.

Preparation of Si anodes

CMC and P1 were respectively used as binders to prepare nanosized Si-based anodes. Firstly, Si powder was mixed with carbon black and the binder in the weight ratio of 6 : 2 : 2. Carbon black was used as a conductive additive. Then the mixture was dissolved in DI water, followed by a vigorous stirring with homogenizer at 1900 rpm for 5 minute to form homogeneous slurry. Finally, the mixed slurry was spread onto a 20 μm thick copper foil and vacuum dried at 150 °C for 12 hours. The coating thickness was ~10 μm.

Electrochemical measurement

The coin half-cells (CR2025) were assembled to test the electrochemical performance of the obtained anodes. Cells were assembled in an Ar-filled glovebox, using 1 M $LiPF_6$-EC/DEC /DMC (1 : 1 : 1, v/v/v) as electrolyte, Li foil as the counter electrode and Celgard 2400 as the separator. Wuhan Landian battery cycler (China) was used to measure battery performance through cyclic voltammetry and charge/discharge cycling at various current density values between cut-off voltage of 0.01 and 1.50 V (vs. Li/Li^+). Cyclic voltammetry (CV) was conducted in cells at the scan rate of 0.2 $mV{\cdot}s^{-1}$ from 10 mV to 1.5 V at room

temperature. Electrochemical impedance spectroscopy (EIS) was measured by applying an oscillating voltage of 5 mV over the frequency ranging from 10^{-2} to 10^{5} Hz. The CV and EIS measurements were carried out on an IM6e electrochemical workstation (Zahner, Germany).

Density functional theory (DFT) calculations:

All DFT calculations were performed using the Vienna ab initio simulation package (VASP) at a PAW-PBE level.[2, 3] Wavefunctions were expanded with plane-waves and the cut off energy was set to be 500 eV. An eight layer 4 × 4 Si-(111) surface (contains 128 Si atoms) with a vacuum layer of about 20 Å was used to simulate the surface of Si anode, and a single layer 6 × 6 graphene was used to simulate the conductive carbon. During the simulation, we fixed the atomic positions of 4-layer Si atoms, while all other atomic positions were fully relaxed until the forces were converged to 0.05 eV/Å. In order to better describe the adsorption interactions between the adsorbed molecules and anode materials, the van der Waals (vdW) corrections of DFT-D3 method with Becke-Jonson damping were included.[4] The adsorption of the –COOH group onto the Si-(111) surface was associated with the bonding of the oxygen atom, which was double bound with the C atom, with the Si atom. Then, the C=O double bond became single bond, and therefore the C-O bond to the -OH group became weakened, resulting in a dissociation of the H atom from the -OH group, which was then adsorbed on the Si surface. On the other hand, Si-O chemical bond might also be formed between the –OCNCCO– group and the Si surface. In this case, The C=O double

bond was also first weakened with the O atom bound with the Si atom, then the bonding state of the C atom became unsaturated.

Materials Characterizations:

The morphology of nano-Si anodes with CMC, P1 and P2 binders before and after cycling was observed by SEM (Hitachi S-4800, Japan). FTIR measurement was recorded on a TENSOR27 spectrometer (Bruker, Germany) from 400 to 4000 cm^{-1} at the resolution of 4 cm^{-1}. The XPS spectra were obtained with ESCALAB250 XPS (Thermo Fisher Scientific, USA) at 2×10^{-9} mbar, using Al K (1486.6 eV) radiation at 15 keV of anode voltage. Peak fitting of the high-resolution data was carried out using the Xpspeak41 software. X-ray diffraction (XRD) experiments were conducted on a Bruker D8 Advance X-ray diffractometer (Bruker Optics, Ettlingen, Germany) with Cu-Ka radiation, operated at 40 kV and 40 mA.

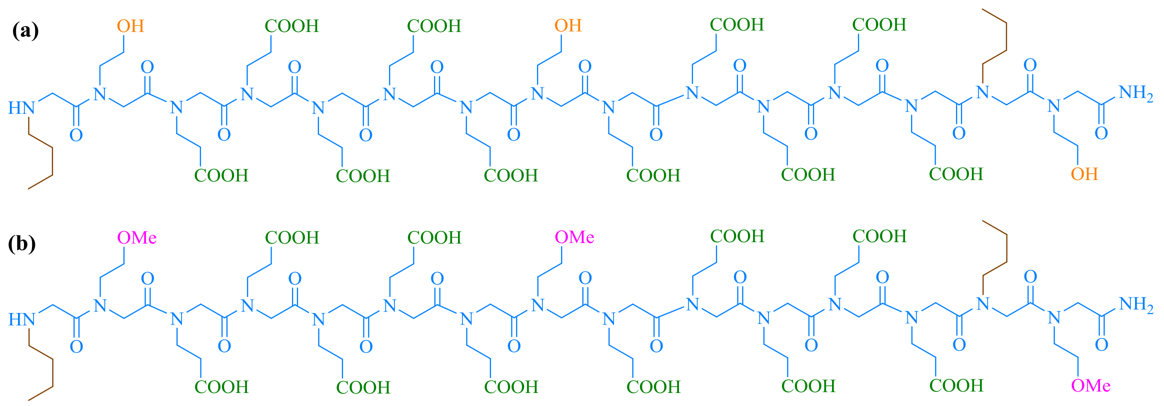

Figure S1. Chemical structure of the sequence-designed P2.

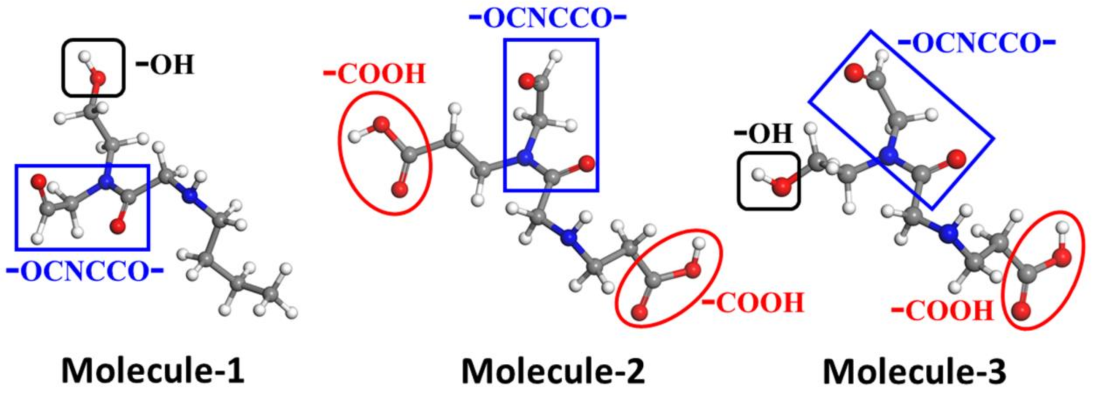

Figure S2. Structures and functional groups of three molecules which are fraction of the Peptoids chain. The oxygen containing functional groups, namely, "–OH", "–COOH" and "–OCNCCO–" are marked with black ellipses, red ellipses and blue rectangles, respectively. The red, gray, blue and white spheres are O, C, N and H atoms, respectively.

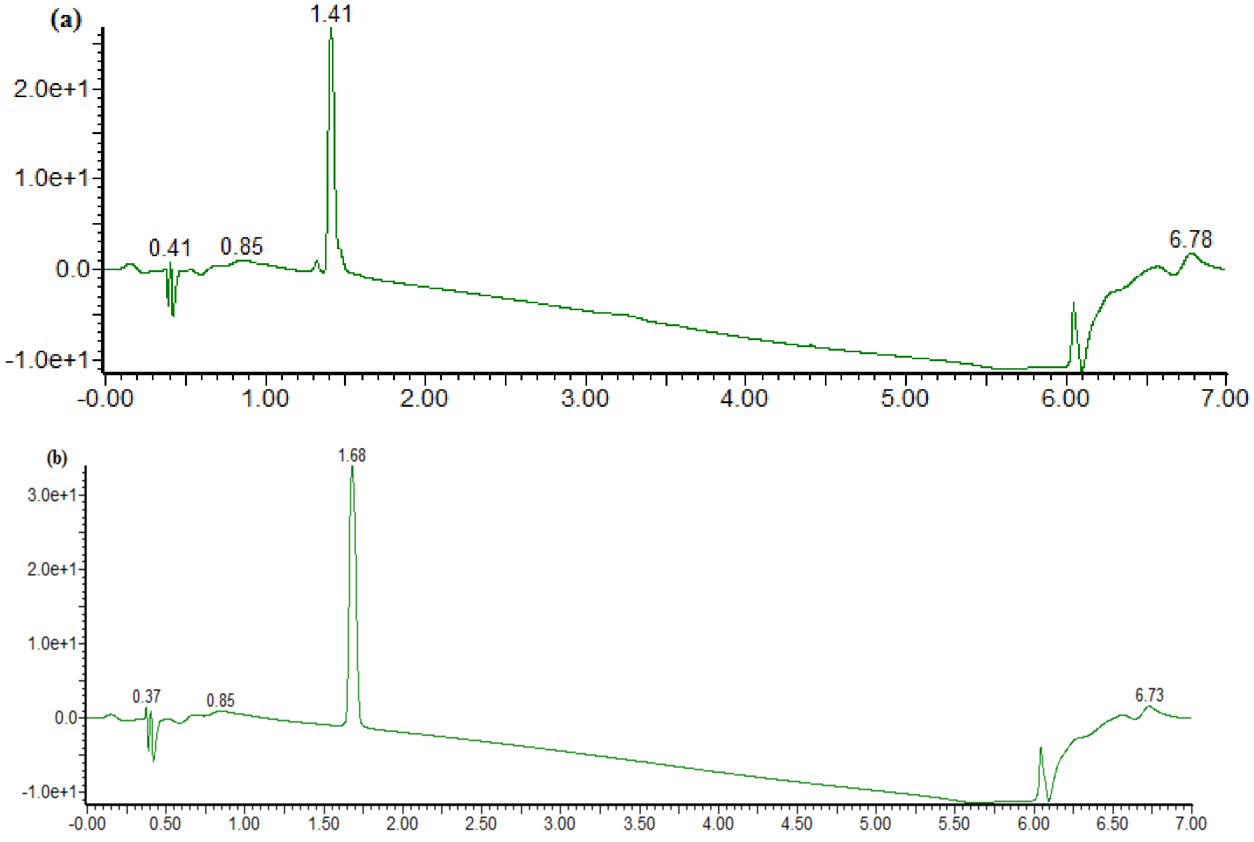

Figure S3. UPLC spectra of (a) P1 and (b) P2 with the gradients of 5~95% CH_3CN in H_2O.

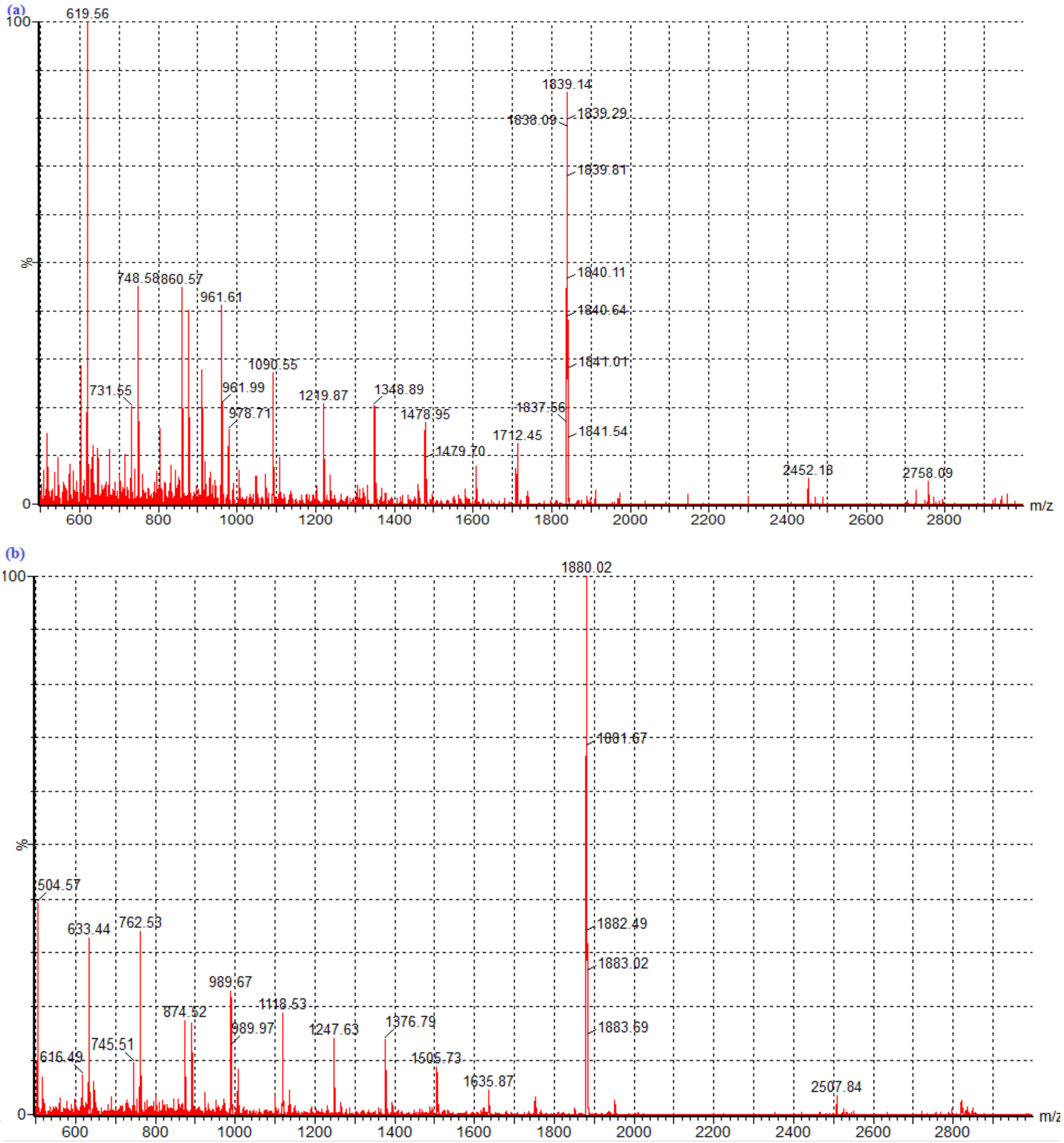

Figure S4. UPLS-MS Spectra of (a) P1 and (b) P2.

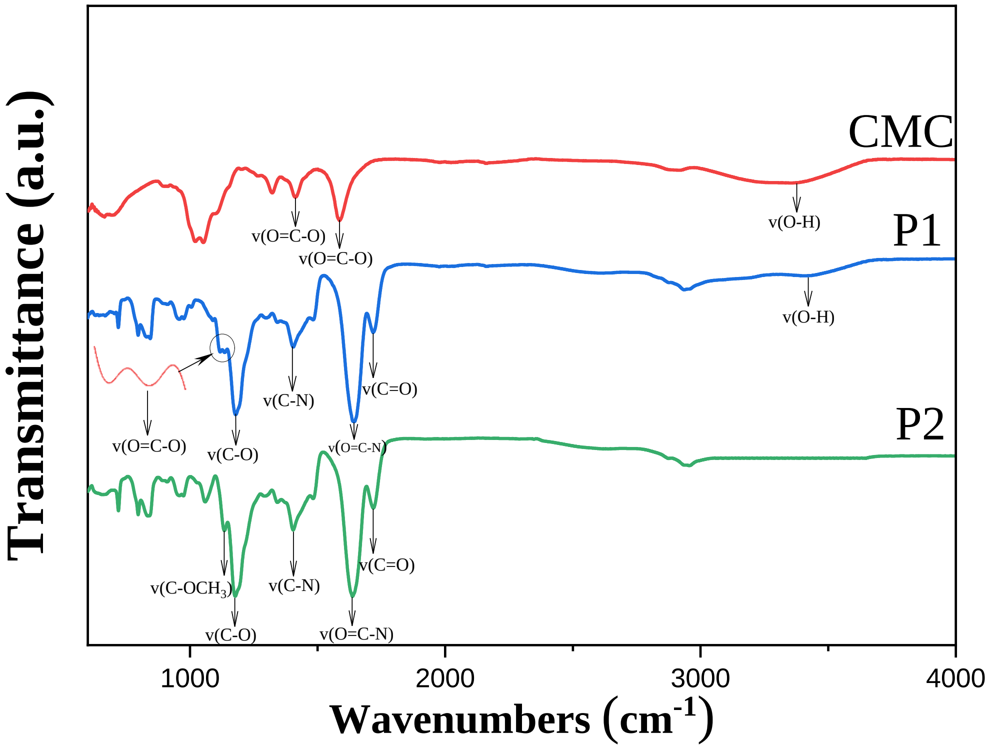

Figure S5. FTIR spectra of pure CMC, P1 and P2.

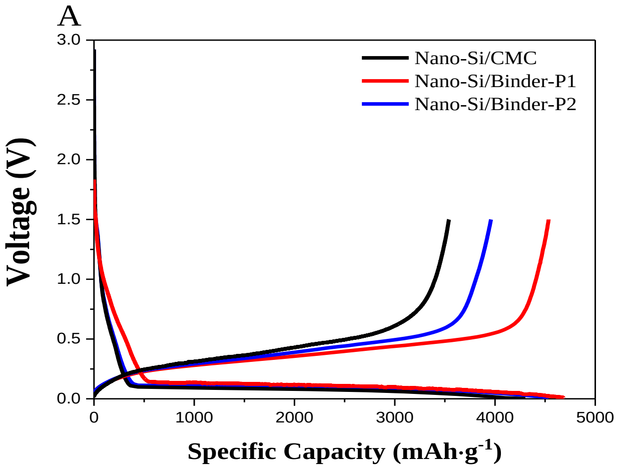
A
Nano-Si/CMC
Nano-Si/Binder-P1
Nano-Si/Binder-P2
Voltage (V)
Specific Capacity (mAh·g-1)

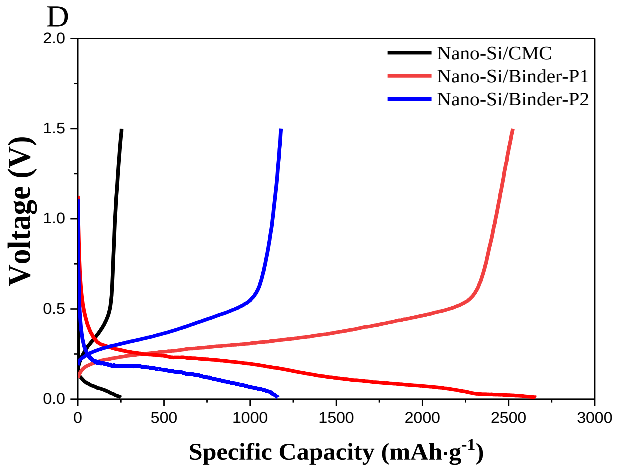
D
Nano-Si/CMC
Nano-Si/Binder-P1
Nano-Si/Binder-P2
Voltage (V)
Specific Capacity (mAh·g-1)

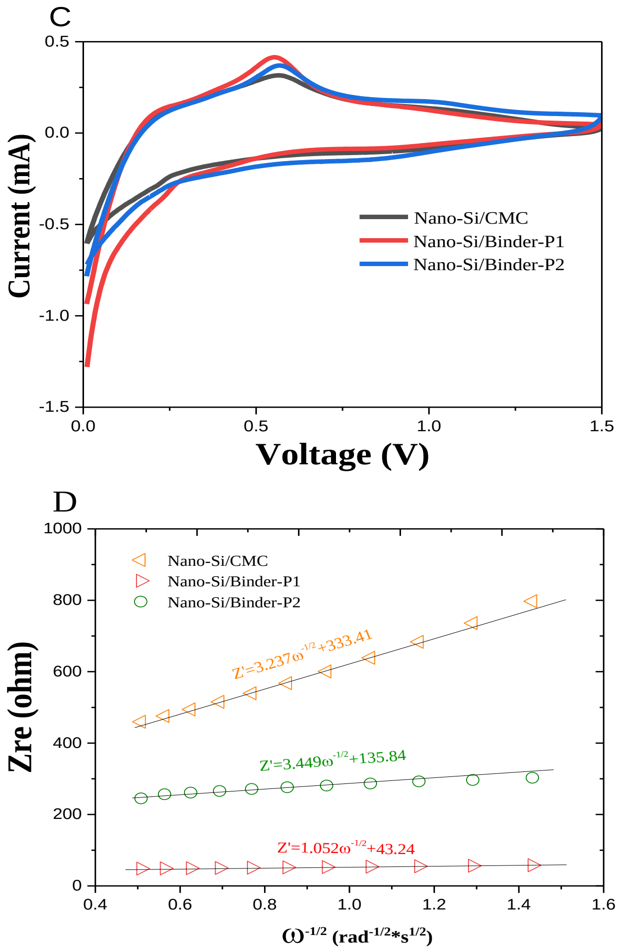

Figure S6. Initial charge-discharge profiles of the nano-Si anodes bound by CMC, P1 and P2 when measured at (A) 1 A·g^{-1}) and (B) 5 C (15 A·g^{-1}), respectively. (C) CV curves of nano-Si electrodes with CMC, P1 and P2 binders at a scan rate of 0.2 mV·s^{-1}. (D) Relationship between Z_{re} and $\omega^{-1/2}$ of the nano-Si electrodes using CMC, P1 and P2 as binders after 100th cycles. The slope (Warburg impedance coefficient σ) is used to calculate the lithium-ion diffusion coefficient.

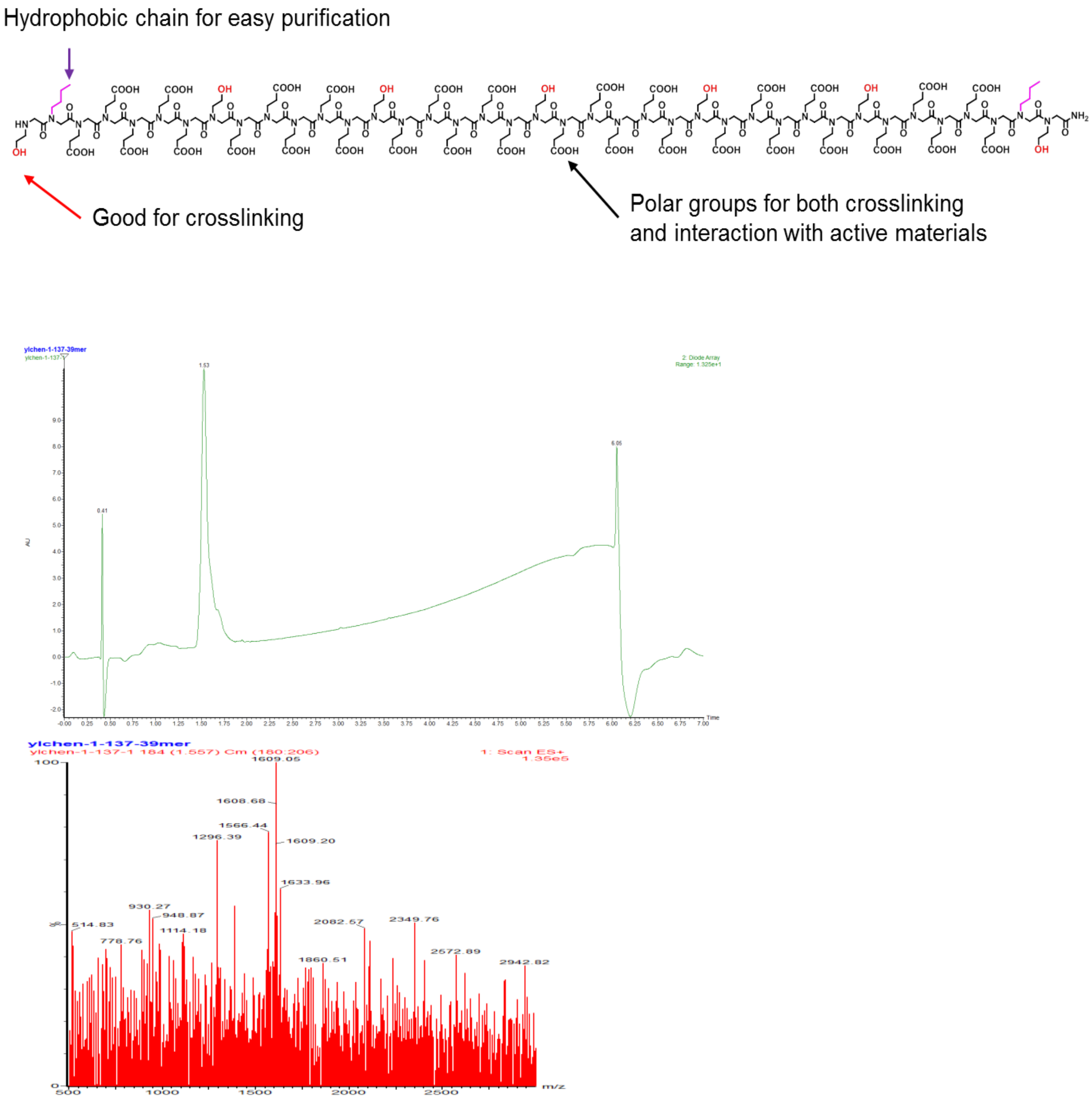

Figure S7. Chemical structure of 39-mer sequence-designed P3 and its UPLC-MS data.

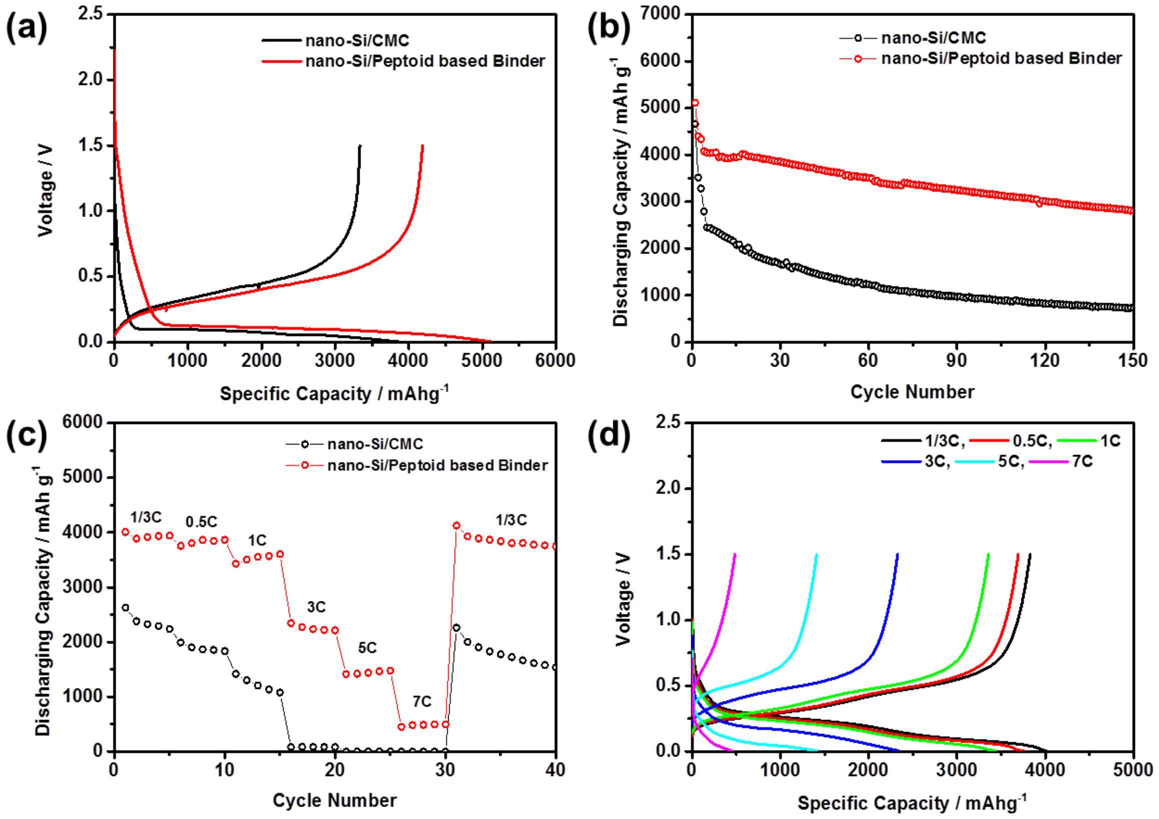

Figure S8. (a) 1st voltage profile of nano-Si anode with CMC and P3 binders, (b) plot of cycle retention at 1/3C after 3 formation cycles at 0.1C, (c) rate capability at various discharging C-rates, and (d) voltage profile of nano-Si anode wth P3 as the binder at 1/3, 0.5, 1, 3, 5, and 7C-rates. For rate capability test, charging C-rate is fixed at 1/3C and cut-off voltage is in the range of 0.005~1.5 V (1C=1500 mA/g).

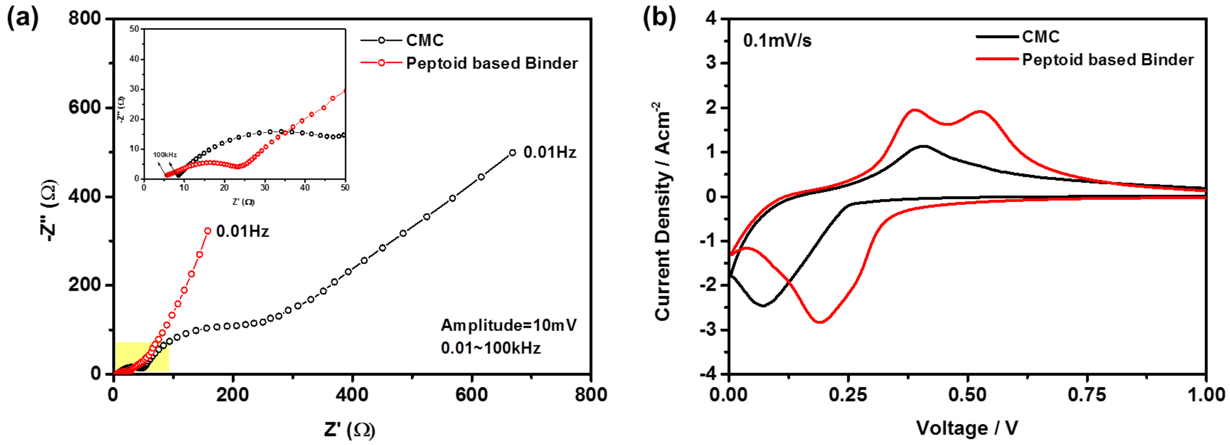

Figure S9. (a) Nyquist plot (inset: the magnified result of yellow highlighted area) and (b) cyclic voltammetry (CV) of CMC and P3-bound anode after 3 formation cycles. The impedance analysis is in the range of 0.01 to 100 kHz with 10 mV amplitude and the scan rate is 0.1 mV/s vs. Li/Li^+ for CV analysis.

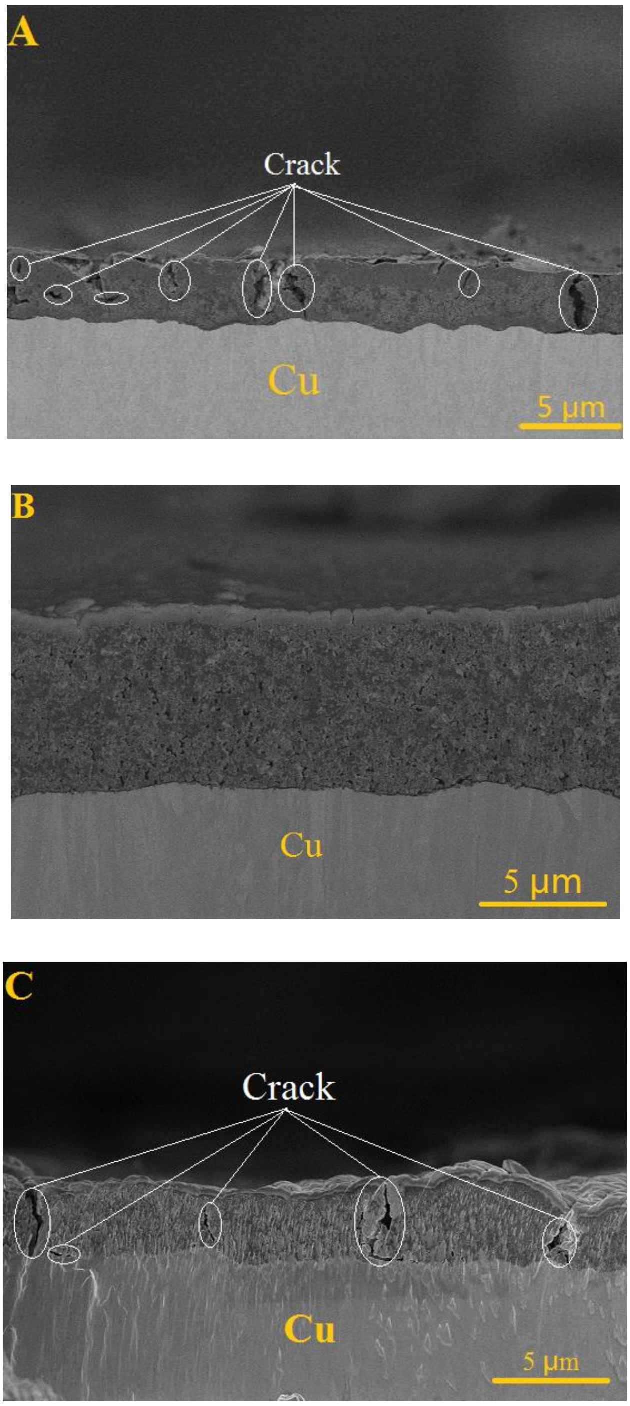

Figure S10. SEM images of the as-prepared Si anodes with (A) CMC, (B) P1 and (C) P2 as binders after 100 cycles.

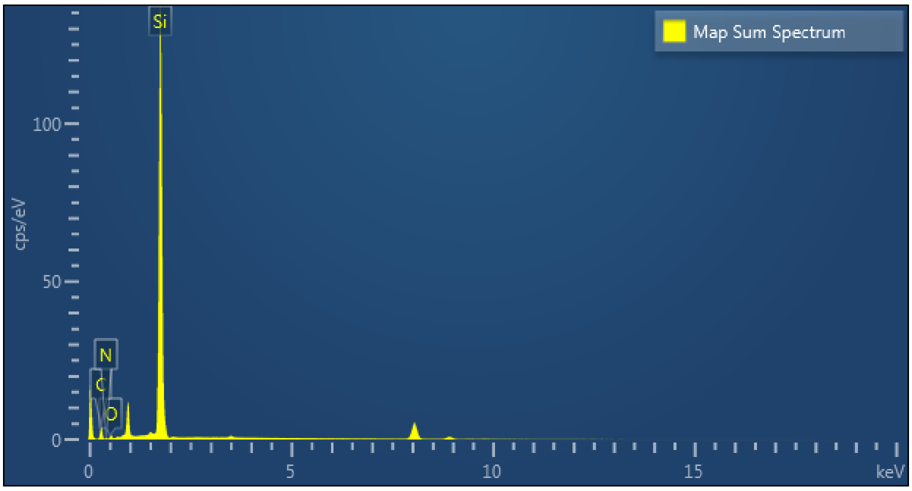

Figure S11. Map Spectrum of Si, C, N and O elements in P1-bound nano-Si anode after 100 cycles.

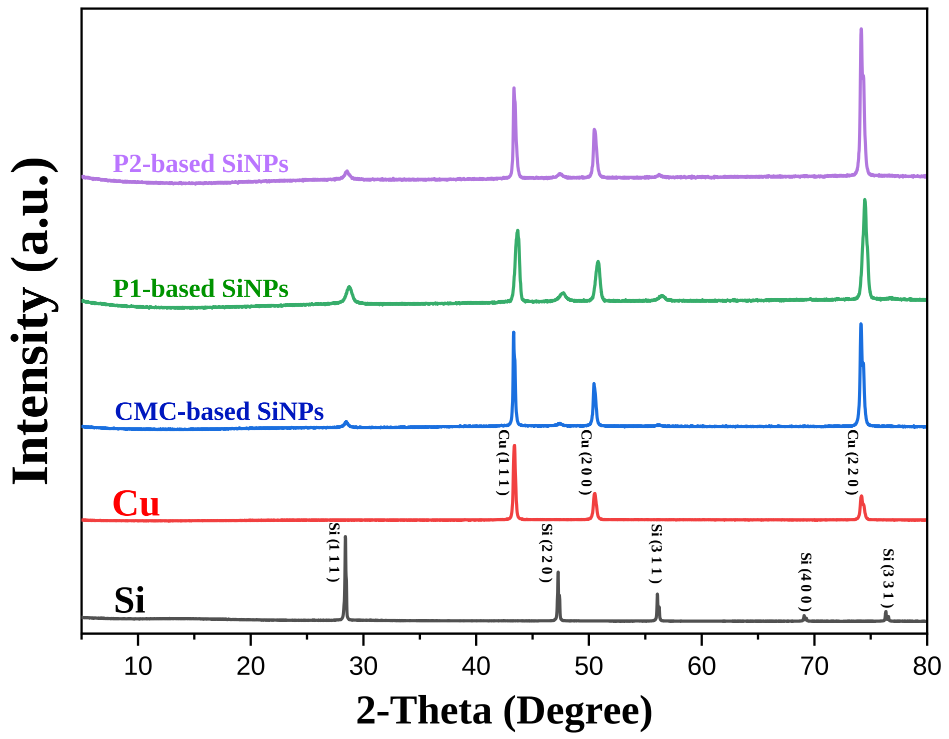

Figure S12. XRD patterns of CMC, P1 and P2 bound Si anodes after 100 cycles.

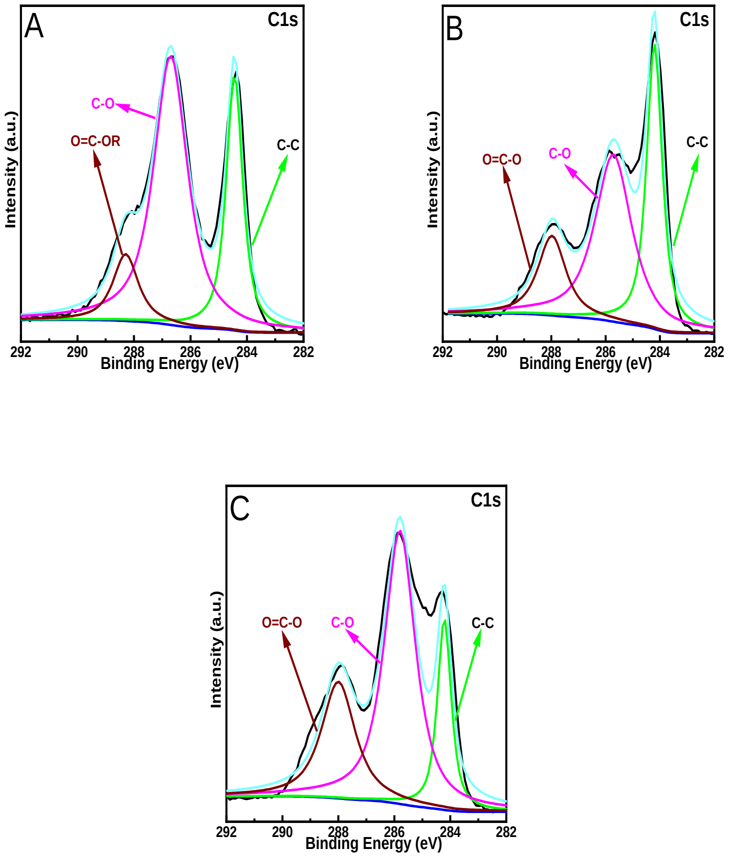

Figure S13. XPS spectra showing the binding energies of C 1s measured for the (a) CMC, (b) P1 and (c) P2 bound anodes following 100 cycles.

Table S1. Adsorption energies E_{ad} (in eV) of the three molecules on the surface of Si (111) and single graphene layer with different adsorption configurations

		Molecule-1	Molecule-2	Molecule-3
Si-111	Physical adsorption	1.19	0.88	1.44
	1 Si-O bond	3.59	3.48	3.61
	2 Si-O bonds		5.54	
graphene		0.79	0.87	0.81

Table S2 Comparison of the electrochemical performances of Si-bound anodes fabricated with different binders.

Binder	Si Particle Size	Electrode Composition	Electrode Mass Loading ($mg·cm^{-2}$)	Electrode Performance ($mAh·g^{-1}$)	Ref.
Peptoid-1	100~200 nm	Si : C : Peptoid-1 = 60 : 20 : 20	~1.5	3110@1 $A·g^{-1}$, 500th	Our work
PR-PAA	2.1 μm	SiMP :PR-PAA :Super P=80 : 10 : 10	1.07	2271@0.1 $A·g^{-1}$, 150th	[5]
c-CMC-CA	50~70 nm	CVSS: CMC-CA:CB = 60: 20 : 20	1.0~1.2	2736@1 $A·g^{-1}$,100th; 1200@5 $A·g^{-1}$,1000th	[6]
c-PAM-0.001	100~200 nm	Si : c-PAM-0.001 : Super P = 70 : 15 : 15	1.0~1.1	2843@0.3 $A·g^{-1}$, 100th	[7]
Karaya Gum (KG)	50~200 nm	Si : Super P : KG = 60 : 20 : 20	0.4~0.5 or 0.8~0.9	2421@1.5 $A·g^{-1}$, 150th; 1000@4.0 $A·g^{-1}$, 1200th	[8]
CGG	300 nm	Si : Super P : CGG = 60 : 20 : 20	0.8	1138@1 $A·g^{-1}$, 200th	[9]
c-Alg-g-PAAm	Not mentioned	Si : C : c-Alg-g-PAAm : Conducting Agent = 19 : 57 : 15 : 9	1.3 ± 0.1	836@0.15 $A·g^{-1}$, 100th	[10]
PAA/PVA(60/ 40)	3.1 μm	Si : C : PAA/PVA = 70 : 16 : 14	2.6 ± 0.2	1200@0.24 $A·g^{-1}$, 100th; 2000@0.40 $A·g^{-1}$, 60th	[11]
PAA-P(HEA-co-DMA)	0.5~3 μm	Si : Super P : Binder = 80 : 10 : 10	2.3	2394@1 $A·g^{-1}$, 220th	[12]
PEFM	≤100 nm	Si : PEFM = 66.6 : 33.3	~ 0.2	1800@7.5 $A·g^{-1}$, 50th 3000@0.375 $A·g^{-1}$, 50th	[13]
Mxenes *Conductive Binder*	~80 nm	nSi : MX-C = 70 : 30	0.9	1602@1.5 $A·g^{-1}$, 280th	[14]
c-PEO-PEDOT: PSS/PEI *Conductive Binder*	180 nm	Si : c-PEO-PEDOT/PEI = 80 : 20	1	2027@1 $A·g^{-1}$, 500th	[15]
PF-COONa *Conductive Binder*	50 nm	Si : PF-COONa = 66.6 : 33.3	0.19	2806@0.42 $A·g^{-1}$, 100th; 999@4.2 $A·g^{-1}$, 1000th	[16]
PEDOT:PSS *Conductive Binder*	50~70 nm	Si: (PEDOT:PSS) = 80 : 20	0.4~1.5	1927 @ 2 $A·g^{-1}$, 100th 2186 @ 0.5 $A·g^{-1}$, 100th	[17]
PPQ *Conductive Binder*	≤ 50 nm	Si: PPQ = 70 : 30	0.8	2823 @ 0.358 $A·g^{-1}$,50th	[18]
PAA/PANIIPN *Conductive Binder*	200 nm	Si: PAA/PANI IPN = 60: 40	~1	2750 @ 0.42 A/g, 100th 2205 @ 0.42 A/g, 300th	[19]

Table S3. Elemental Composition of P1-bound nano-Si anode after 100 cycles.

Element	Line Type	Apparent Concentration	k Ratio	Wt%	Wt% Sigma	Atomic %	Standard Label	Factory Standard
C	K series	4.20	0.04196	41.49	0.33	60.32	C Vit	Yes
N	K series	1.01	0.00180	2.21	0.33	2.75	BN	Yes
O	K series	1.75	0.00590	4.09	0.11	4.46	SiO2	Yes
Si	K series	67.27	0.53304	52.22	0.32	32.47	SiO2	Yes
Total:				100.00		100.00		

Schemes

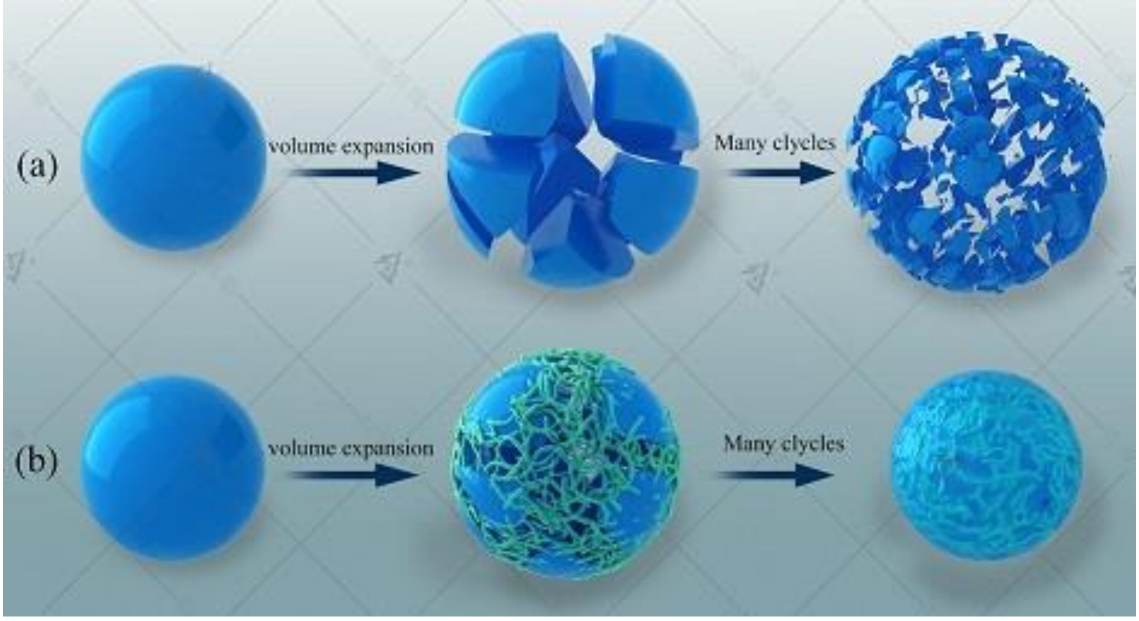

Scheme S1. Proposed mechanism of (a) normal binders like PVDF and (b) P1 binder for nano-Si anode.

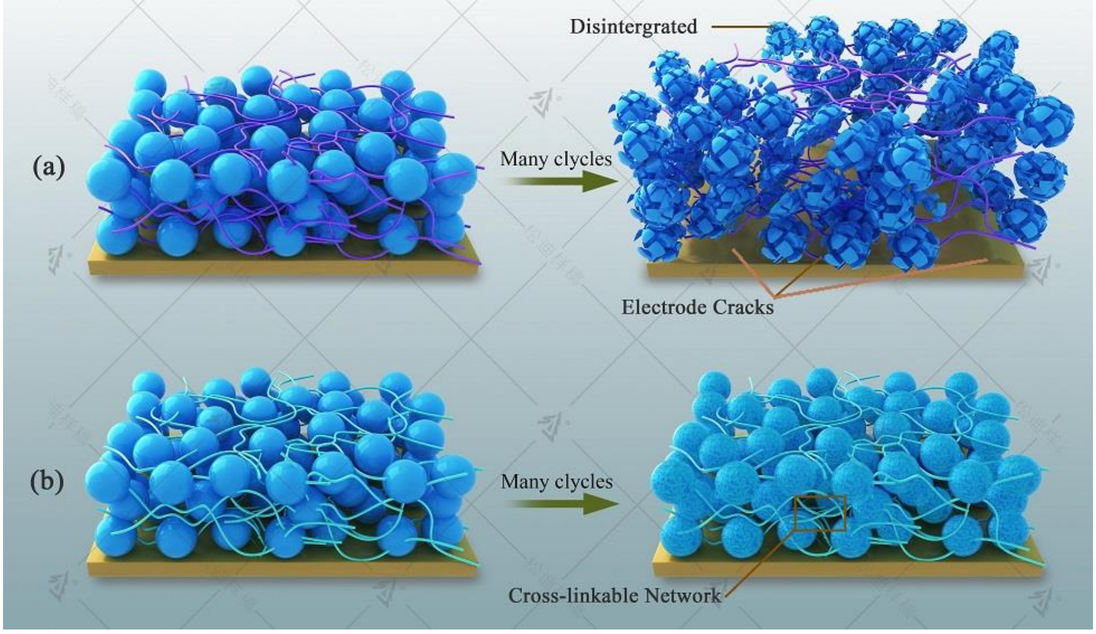

Scheme S2. Schematic drawing of the proposed mechanism of (a) CMC and (b) P1 binders for nano-Si anodes.

References

[1] a) Kresse, G.; Furthmüller, J. Efficient Iterative Schemes for AB Initio Total-energy Calculations Using a Plane-wave Basis Set. *Physical Review B* **1996**, *54*, 11169-11186; b) R. N. Zuckermann, J. M. Kerr, S. B. H. Kent, W. H. Moos, *J. Am. Chem. Soc.* **1992**, *114*, 10646.

[2] Perdew, J. P.; Chevary, J. A.; Vosko, S. H.; Jackson, K. A.; Pederson, M. R.; Singh, D. J.; Fiolhais, C. Atoms, Molecules, Solids, and Surfaces: Applications of the Generalized Gradient Approximation for Exchange and Correlation. *Physical Review B* **1992**, *46*, 6671-6687.

[3] Blöchl, P. E. Projector Augmented-wave Method. *Physical Review B* **1994**, *50*, 17953-17979.

[4] Grimme, S.; Ehrlich, S.; Goerigk, L. Effect of the Damping Function in Dispersion Corrected Density Functional Theory. *J. Comp. Chem.* **2011**, *32*, 1456-1465.

[5] S. Choi, T. W. Kwon, A. Coskun, J. W. Choi, *Science* **2017**, *357*, 279.

[6] Y. J. Liu, Z. X. Tai, T. F. Zhou, V. Sencadas, J. Zhang, L. Zhang, K. Konstantinov, Z. P. Guo, H. K. Liu, *Adv. Mater.* **2017**, *29*, 1703028.

[7] X. Y. Zhu, F. Zhang, L. Zhang, L. Y. Zhang, Y. Z. Song, T. Jiang, S. Sayed, C. Lu, X. G. Wang, J. Y. Sun, Z. F. Liu, *Adv. Funct. Mater.* **2018**, *28*, 1705015.

[8] Y. T. Bie, J. Yang, Y. N. Nuli, J. L. Wang, *J. Mater. Chem. A* **2017**, *5*, 1919.

[9] S. M. Hu, Z. X. Cai, T. Huang, H. B. Zhang, A. S. Yu, *ACS Appl. Mater. Interfaces* **2019**, *11*, 4311.

[10] B. Gendensuren, E. S. Oh, *J. Power Sources* **2018**, *384*, 379.

[11] Q. Y. Huang, C. Y. Wan, M. Loveridge, R. Bhagat, *ACS Appl. Energy Mater.* **2018**, *1*, 6890.

[12] Z. X. Xu, J. Yang, T. Zhang, Y. N. Nuli, J. L. Wang, S. I. Hirano, *Joule* **2018**, *2*, 950-961.

[13] M. Wu, X. Xiao, N. Vukmirovic, S. Xun, P. K. Das, X. Song, P. Olalde-Velasco, D. Wang, A. Z. Weber, L.-W. Wang, V. S. Battaglia, W. Yang, G. Liu, *J. Am. Chem. Soc.* **2013**, *135*, 12048.

[14] C. F. Zhang, S. H. Park, A. Seral-Ascaso, S. Barwich, N. McEvoy, C. S. Boland, J. N. Coleman, Y. Gogotsi, V. Nicolosi, *Nature Commun.* **2019**, *10*, 849.

[15] W. W. Zeng, L. Wang, X. Peng, T. F. Liu, Y. Y. Jiang, F. Qin, L. Hu, P. K. Chu, K. F. Huo, Y. H. Zhou, *Adv. Energy Mater.* **2018**, *8*, 1702314.

[16] D. Liu, Y. Zhao, R. Tan, L. L. Tian, Y. D. Liu, H. B. Chen, F. Pan, *Nano Energy* **2017**, *36*, 206.

[17] T. M. Higgins, S. H. Park, P. J. King, C. F. Zhang, N. McEvoy, N. C. Berner, D. Daly, A. Shmeliov, U. Khan, G. Duesberg, V. Nicolosi, J. N. Coleman, *ACS Nano* **2016**, *10*, 3702.

[18] S. M. Kim, M. H. Kim, S. Y. Choi, J. G. Lee, J. Jang, J. B. Lee, J. H. Ryu, S. S. Hwang, J. H. Park, K. Shin, Y. G. Kim, S. M. Oh, *Energ Environ. Sci.* **2015**, *8*, 1538.

[19] X. H. Yu, H. Y. Yang, H. W. Meng, Y. L. Sun, J. Zheng, D. Q. Ma, X. H. Xu, *ACS Appl. Mater. Inter.* **2015**, *7*, 15961.
